# Soil–plant interactions modulated water availability of Swiss forests during the 2015 and 2018 droughts

**DOI:** 10.1111/gcb.16332

**Published:** 2022-07-24

**Authors:** Katrin Meusburger, Volodymyr Trotsiuk, Paul Schmidt‐Walter, Andri Baltensweiler, Philipp Brun, Fabian Bernhard, Mana Gharun, Raphael Habel, Frank Hagedorn, Roger Köchli, Achilleas Psomas, Heike Puhlmann, Anne Thimonier, Peter Waldner, Stephan Zimmermann, Lorenz Walthert

**Affiliations:** ^1^ Swiss Federal Institute for Forest Snow and Landscape Research (WSL) Birmensdorf Switzerland; ^2^ Agrometeorological Research Center German Weather Service (DWD) Braunschweig Germany; ^3^ Department of Environmental Systems Science ETH Zürich Zürich Switzerland; ^4^ Department of Geosciences University of Münster Münster Germany; ^5^ Department of Soil and Environment Forest Research Institute Baden Württemberg Freiburg Germany

**Keywords:** climate impact, European summer drought, physiological drought, plant‐available water storage capacity, root water uptake, water balance

## Abstract

Central Europe has been experiencing unprecedented droughts during the last decades, stressing the decrease in tree water availability. However, the assessment of physiological drought stress is challenging, and feedback between soil and vegetation is often omitted because of scarce belowground data. Here we aimed to model Swiss forests' water availability during the 2015 and 2018 droughts by implementing the mechanistic soil‐vegetation‐atmosphere‐transport (SVAT) model LWF‐Brook90 taking advantage of regionalized depth‐resolved soil information. We calibrated the model against soil matric potential data measured from 2014 to 2018 at 44 sites along a Swiss climatic and edaphic drought gradient. Swiss forest soils' storage capacity of plant‐available water ranged from 53 mm to 341 mm, with a median of 137 ± 42 mm down to the mean potential rooting depth of 1.2 m. Topsoil was the primary water source. However, trees switched to deeper soil water sources during drought. This effect was less pronounced for coniferous trees with a shallower rooting system than for deciduous trees, which resulted in a higher reduction of actual transpiration (transpiration deficit) in coniferous trees. Across Switzerland, forest trees reduced the transpiration by 23% (compared to potential transpiration) in 2015 and 2018, maintaining annual actual transpiration comparable to other years. Together with lower evaporative fluxes, the Swiss forests did not amplify the blue water deficit. The 2018 drought, characterized by a higher and more persistent transpiration deficit than in 2015, triggered widespread early wilting across Swiss forests that was better predicted by the SVAT‐derived mean soil matric potential in the rooting zone than by climatic predictors. Such feedback‐driven quantification of ecosystem water fluxes in the soil–plant‐atmosphere continuum will be crucial to predicting physiological drought stress under future climate extremes.

## Graphical abstract

Swiss early‐wilting occurrence in August 2018 (adapted after Brun et al., 2020) was best explained by mean soil matric potential in the rooting zone (ψs) and actual to potential transpiration Ta/Tp. Mean soil matric potential in the rooting zone and actual to potential transpiration in pixels with and without early wilting are significantly different and indicate early wilting thresholds.

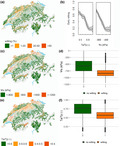

## 1 INTRODUCTION

In 2003, 2015, and 2018, Central Europe experienced the most severe droughts over the past 2100 years (Büntgen et al., 2021). These droughts impressively showed that water availability is vital to forest health (Senf et al., 2020) as it impacts ecosystem functioning and productivity (Babst et al., 2010; Beer et al., 2010; Jung et al., 2017; Nussbaumer et al., 2020; Trotsiuk et al., 2020) and the runoff and drainage from forests (blue water) that may refill ground and surface water resources. Water availability via plants further feedbacks on surface temperature and vapour pressure deficit (VPD) and has a dominant role in separating energy and water fluxes, modulating the carbon sink strength (Gao et al., 2021; Granier et al., 2007; Humphrey et al., 2021). Limited water supply triggers a cascade of physiological responses, such as stomatal closure, reduced water and nutrient uptake, downregulation of photosynthesis, early wilting, crown dieback, and mortality, as observed in 2018 in Germany, Austria, and Switzerland (Obladen et al., 2021; Schuldt et al., 2020; Walthert et al., 2021). So far, several studies have focused on the impacts of recent droughts on carbon assimilation using eddy covariance measurements and remote sensing data (Gharun et al., 2020; Ramonet et al., 2020; Stocker et al., 2019; Thompson et al., 2020; Wang et al., 2020), mapping of early wilting via anomalies in remote sensing‐derived vegetation indices (Brun et al., 2020; Schuldt et al., 2020; Sturm et al., 2022), characterizing these droughts in terms of climatic characteristics (Buras et al., 2020) or in terms of its remote‐sensing derived water mass deficit (Boergens et al., 2020), and evapotranspiration anomalies (Ahmed et al., 2021). Only a few studies have evaluated these droughts with mechanistic models (Mastrotheodoros et al., 2020; Moravec et al., 2021), targeting hydrological impacts rather than physiological drought stress.

Vegetation and, in particular, trees modulate the water balance by stomatal activity (Ewers et al., 2005) and are returning water to the atmosphere (‘green water’) by transpiration. Trees either follow a conservative, drought‐avoiding water‐use strategy and effectively control transpiration by stomatal regulation in response to atmospherically or soil drought (Teuling et al., 2010), or sustain high stomata conductance and water consumption at high atmospheric demand to maintain assimilation, at the risk of severe xylem embolism (Massmann et al., 2019). Soils largely determine whether water is available to plants due to their ability to retain precipitation, store water, and host plant roots. Consequently, they mediate between a meteorological drought (lack of precipitation, high air temperature) and a physiological drought (water deficiency, not covering plant needs). The amount of water available to plants depends on soil properties (e.g. stone content, texture, bulk density) and is quantified by the water retention curve, the relationship between the volumetric soil water content, *θ*, and the soil matric potential, *ψ*_s_. Unlike soil water content, the soil matric potential is a comparable measure of water availability for plant roots across different soil characteristics and is directly related to plant water potential (Novick et al., 2022; Steppe, 2018) and distinct levels of drought stress, as was shown for beech (Walthert et al., 2021). Furthermore, root distribution and related root water uptake (RWU) are critical constraints for the plant water supply. Finally, the spatiotemporal variability of the soil water availability needs to be matched with the root depth distribution to derive the development of drought stress over time.

Commonly applied drought indices are predominantly based on meteorological quantities, such as the standardized precipitation index and the standardized precipitation evapotranspiration index (Vicente‐Serrano et al., 2010). Being fully empiric, they implicitly assume water storage in the soils during rainfall and water usage for evapotranspiration during (precedent) sunshine. However, physiological drought intensity depends on plant physiological and morphological traits and their interference with the soil and atmosphere. Drought indices more explicitly related to soil moisture or physiological drought typically rely on numerical models such as the German drought monitor (Samaniego et al., 2018). While the Palmer Drought Severity Index (Palmer, 1965) takes the available water storage capacity (AWC) as a simple bucket besides temperature and precipitation into account, indices based on hydrological models like the Evapotranspiration Deficit Index or the Soil Moisture Deficit Index have been shown to be superior in predicting physiological drought (Schwärzel et al., 2009; Sepulcre‐Canto et al., 2014; Speich, 2019; Wu et al., 2021). An even closer link to physiological drought is expected from the transpiration deficit (*T*_d_ also referred to as ED), expressed as the difference or ratio between actual (*T*_a_) and potential transpiration (*T*_p_) (Leuning, 1995; Vicente‐Serrano et al., 2015; Zierl, 2001) or the soil matric potential (Schmidt‐Walter et al., 2019).

Mechanistic soil‐vegetation‐atmosphere‐transport (SVAT) models, in contrast to bucket‐type models, can account for interactions in the soil–plant‐atmosphere continuum. They balance belowground water supply with aboveground water demand to simulate water availability and physiological drought (Federer, 1979; Schmidt‐Walter et al., 2020) and may improve urgently needed understanding of ecosystem responses to drought (Xu et al., 2013). However, the application of SVAT models at the regional scale is often impeded by low data availability and the inability to constrain model parameters. While the availability of aboveground vegetation characteristics is rapidly increasing thanks to remote sensing techniques, belowground information is still often limited. For Switzerland, regionalized information on depth‐resolved soil properties and maximum potential rooting depth (mrd) was recently made available (Baltensweiler et al., 2021). With Swiss‐wide soil matric potential measurements, including the two major droughts (2015 and 2018), the application of a physically based SVAT model became feasible.

In our study, we implemented the SVAT model LWF‐Brook90 for the forested area of Switzerland using >160,000 days of soil matric potential measurements for the model calibration. We aimed at a feedback‐driven separation between green and blue water fluxes in the Swiss forest areas and to assess the impact of 2015 and 2018 droughts on these water fluxes. A second objective was to delineate critical thresholds of drought intensity/water availability to improve predictions of early wilting.

## 2 MATERIALS AND METHODS

In the first step, we mapped the AWC of forest soils using pedotransfer functions (PTFs) to predict the soil hydraulic parameters of the water retention curve based on regionalized soil parameters (Baltensweiler et al., 2021). Then, the dynamic filling and emptying of the AWC was simulated with the calibrated SVAT LWF‐Brook90 model. Finally, we derived the water budget and drought indices from the model application. The latter were used to predict the 2018 early wilting pattern with a general additive model (GAM) and to derive early wilting thresholds.

### 2.1 SVAT model: LWF‐Brook90

The process‐based SVAT model LWFBrook90 (Hammel & Kennel, 2001), recently implemented in an R environment (Schmidt‐Walter et al., 2020), was used for water balance simulations (package *LWFBrookR* version 0.4.5). LWF‐Brook90 is a modification of the well‐known Brook90 model (Federer, 2002), which simulates daily transpiration and interception from a single layer plant canopy (big leaf) and soil water fluxes through the soil profile. The interception is calculated following Rutter et al. (1972), and the simulation of snow accumulation and melt relies on a traditional degree‐day method for estimating snow energy balance.

Shuttleworth and Wallace (1985) modification to Penman–Monteith potential evapotranspiration separates transpiration and soil evaporation. With Federer's method, potential transpiration is reduced to actual transpiration (Federer et al., 2003). On a sub‐daily basis (assuming that the potential transpiration rate varies as a half‐sine wave), actual transpiration is the minimum of potential transpiration and the water supply rate on the soil‐to‐leaf pathway, taking into account root density distribution, rhizosphere and plant root and xylem resistance, and the critical leaf water potential at which stomata close. RWU is described by the single root model (Cowan, 1965; Hillel & van Bavel, 1976). Assuming a uniform radial root distribution around the stem allows for estimating the rooting zone. Here we parameterized relative root length densities of the soil layers from the cumulative proportion of roots derived by the model after Gale and Grigal (1987). The maximum rooting depth and the shape parameter *beta* were used as fitting parameters, with maximum rooting depth and depth distribution observed in the soil profiles at 44 sites as prior parameters. RWU decreases with decreasing hydraulic conductivity of the rooting zone, which is assumed to correspond to the hydraulic conductivity of the bulk soil. As water uptake is computed for each soil layer, roots with access to moist soil layers enable compensation of water uptake to account for the transpiration demand.

The water movement in the soil is modelled by numerically solving the Richards equation using the Mualem–van Genuchten hydraulic parameters (Mualem, 1976; van Genuchten, 1980). The Mualem–van Genuchten parameters were predicted based on regionalized and depth‐resolved soil properties (described below) with two PTFs, namely Wessolek et al. (2009) and Puhlmann and von Wilpert (2011). Organic forest floor layers were not considered in this study.

The dynamic of the leaf area index (LAI) was simulated with the R package *vegperiod* version 0.3.1 (Nuske, 2017), where dates of budburst were calculated with the degree‐day approaches described in Menzel (1997) and the end of the vegetation period when LAI starts to decline with the method of von Wilpert (1990). The LAI seasonality was delineated with the b90 method of the *LWFBrook90R* package.

### 2.2 Initial and forcing data for model simulations

The SVAT model was applied at the site and Swiss scale (forested sites, 500 m spatial resolution). The SVAT model was forced by the daily meteorological data (air temperature [mean, max, min], precipitation, relative humidity, global radiation, vapour pressure, and wind speed) derived from MeteoTest (MeteoTest, 2020) for both application scales from 2013 to 2019 (250 m spatial resolution). The year 2013 was solely used to dampen transient effects of uncertain initial conditions and was excluded from further analysis.

At the site level used for calibration and validation, measured LAI, tree height, tree species, and elevation and slope angle were measured in the field, and the soil properties from a soil profile were used as input data. LAI calculation was based on hemispherical photos taken in the middle of the vegetation periods in 2014 and 2015, representing the LAI max of each stand. The soil hydraulic parameters were derived from the soil properties texture, soil organic carbon, bulk density, and gravel content (for the PTF of Puhlmann & von Wilpert, 2011), and texture and gravel content (for Wessolek et al., 2009) per soil horizon.

For the spatial model application, LAI (500 m pixel size) was derived from a Moderate Resolution Imaging Spectroradiometer (MODIS) over the years 2015–2018 (Justice et al., 2002), tree height from the 1 m spatial resolution vegetation height model (Ginzler & Hobi, 2016) and tree type (fraction of broadleaved versus needle‐leaved trees) at 3 m spatial resolution from a Swiss broadleaf distribution map (Waser et al., 2017). A 50% broadleaf abundance threshold was used to classify deciduous and coniferous forests. Elevation and slope angle were derived from the digital elevation model DHM25 of Swisstopo with a spatial resolution of 25 m.

The spatial SVAT modelling relies on recently published maps of soil properties (Baltensweiler et al., 2021) with information on clay, sand, gravel, and C_org_ contents and fine earth density for the following soil depths: 0–5, 5–15, 15–30, 30–60, 60–100, and 100–200 cm. In addition, the mrd was also spatially available. These maps (25 m spatial resolution) are based on random forest models created from 2071 forest soil profiles. The models were evaluated against an external, independent data set and achieved an *R* ^2^ of 0.21–0.49 for texture (clay, sand, and gravel contents) across all soil depths. While for fine earth density, the *R* ^2^ varied between 0.51 and 0.64 across all depth intervals, soil organic carbon content was more difficult to predict (*R* ^2^ = 0.19–0.32). The mrd map was also validated with the external dataset and achieved an *R* ^2^ of 0.19. Finally, we applied the PTFs introduced above to all depth layers to generate maps of plant‐ AWC (between −6.3 and −1585 kPa) and gravitational water storage capacity (GWC, between 0 and −6.3 kPa). Uncertainties of the spatial input soil layers were propagated through the calculations of AWC and GWC by sampling 1000 within the 95% confidence interval of each pixel of sand, C_org_, and gravel contents taking the covariance of sand and clay into account. Then, uncertainties of cumulated AWC and GWC down to a specific depth (1 m, 2 m, mrd) were calculated by the additive formula for error propagation.

Despite higher resolution input data, a spatial resolution of 500 m was selected for efficient SVAT simulation. The data were aggregated by bilinear resampling. All analyses and plotting have been carried out in Rx64 4.1 using the packages *sf (1.0–1)*, *sp (1.4–5)*, *raster (3.4–13), hydroGOF (0.4–0), dplyr (1.0.7), tidyr (1.1.3), lubridate (1.7.10), purr (0.3.4), viridis (0.6.1), data. table (1.14.0)* and *ggplot2 (3.3.5)*.

### 2.3 Sites with measurements

**FIGURE 1:**
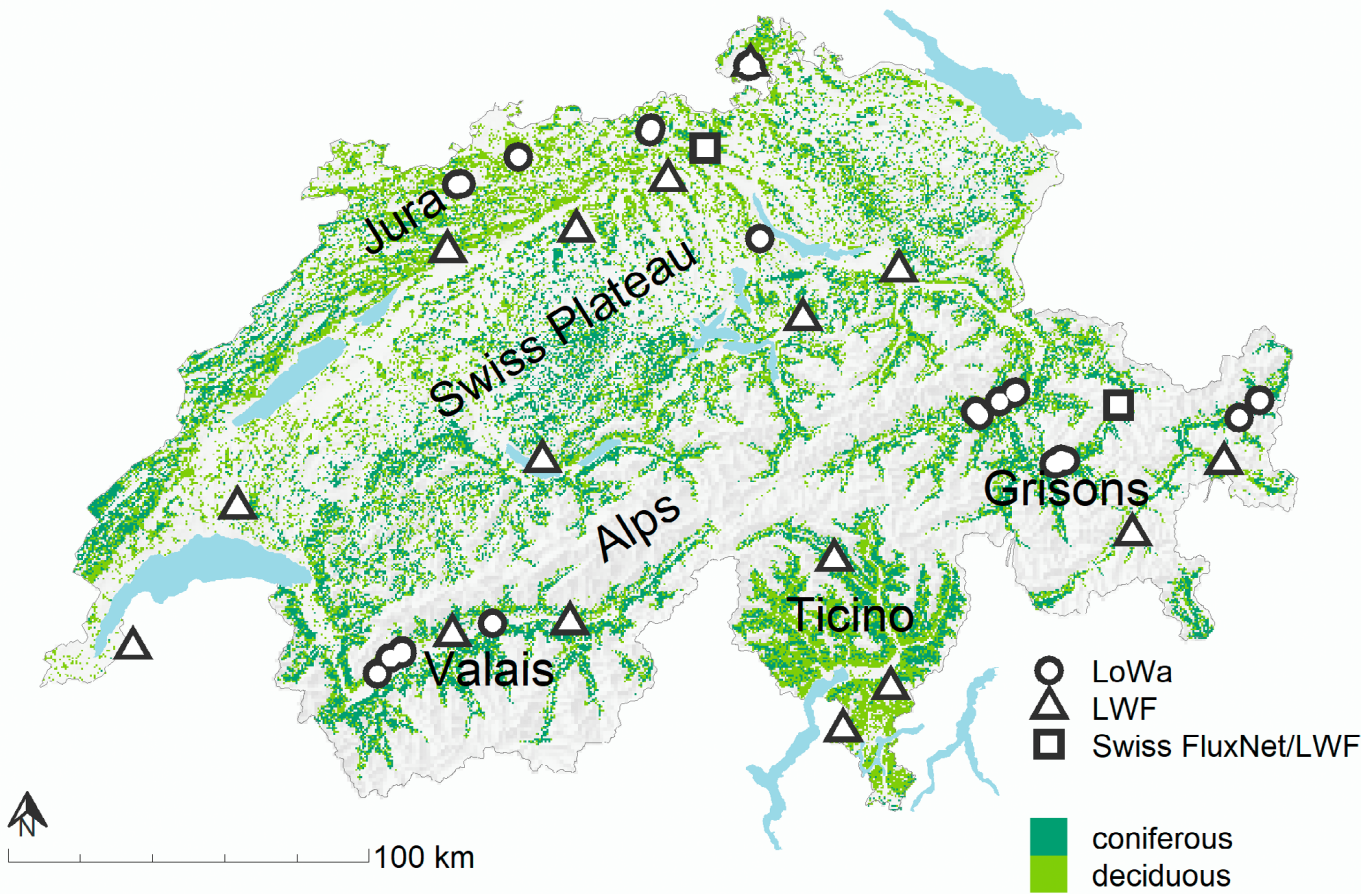
Location of sites with soil matric potential measurements used for model calibration and validation along a Swiss climatic and edaphic drought gradient (*n* = 44, LoWa). For plausibility checks, we used eddy covariance measurements from two Swiss FluxNet sites that are also part of the Swiss Long‐term Forest Ecosystem Research Programme (LWF). From 11 LWF sites, throughfall measurements were used for model plausibilization.

Starting in 2013, soil matric potential was measured at 44 sites (Figure 1) located on a climatic‐edaphic drought gradient across Switzerland (LoWa network). The sites follow a precipitation gradient (between 650 and 1370 mm mean annual precipitation, MAP) from the Central Valais (*n* = 17 with 744 ± 55 mm MAP), Eastern Grisons (*n* = 10 with 870 ± 94 mm MAP), Eastern Jura (*n* = 10 with 957 ± 120 mm MAP), to the Central Grisons (*n* = 7 with 1003 ± 102 mm MAP). Mean annual temperatures range between 4.9 and 9.9°C. Measured forests include beech (*n* = 10), spruce (*n* = 3), pine (*n* = 13), oak (*n* = 8), and mixed stands of spruce with pine (*n* = 5) and oak with beech (*n* = 5). According to the World Reference Base for Soil Resources, soil types are mainly Leptosols and Cambisols of varying soil depth and stone content. The sites cover an elevation range from 455 to 1495 m asl with a mean elevation of 1075 m asl for coniferous and 717 m asl for deciduous stands.

MPS‐2 probes (MeterGroup, Decagon Devices) were used for soil matric potential measurements that were temperature corrected according to Walthert and Schleppi (2018). The range of accurate measurements for MPS‐2 probes is from −9 kPa to about −1000 kPa (corresponding to pF 1.95 and 4, respectively). Probes were installed at 20 and 80 cm depth in all soils (only in two sites, the lower installation depth was 50 and 70 cm, respectively). For 17 sites, additional sensors were installed at a third (between 110 and 150 cm) and a fourth soil depth (between 140 and 200 cm). The soil matric potential data were used for the sensitivity analysis, calibration and validation of model parameters.

For model plausibilization, we used throughfall‐, LAI data (Thimonier et al., 2019, 2010) measured at 11 LWF sites, and above‐canopy actual evapotranspiration (ET_a_) measured at two Swiss FluxNet sites, which are also part of the Swiss Long‐term Forest Ecosystem Research (LWF) programme network (Figure 1).

### 2.4 Sensitivity analysis, calibration, and validation procedure

Generally, model sophistication is paralleled by introducing more adjustable parameters, increasing the problem of equifinality, where different parameter sets may produce similar model outputs. The calibration procedure we applied improves model reliability and reduces the equifinality problem (Beven & Kirkby, 1979). The model calibration was targeted at finding the best combination of model parameters with respect to the depth‐resolved matric potential measurements. The goal was (i) to derive two calibrated parameter sets for deciduous (oak and beech) and coniferous (pine and spruce) forests and (ii) to assess the model performance and reliability with input data that is not spatially aggregated to 500 m raster cell resolution.

Before the model calibration, regional sensitivity analysis via Monte Carlo Filtering (Hornberger & Spear, 1981) was conducted to identify sensitive model parameters that significantly improved the agreement between measured and simulated soil matric potential (kPa). A combined objective function *cof* using Nash–Sutcliffe efficiency (NSE) and Kling Gupta efficiency (KGE) was used
(1)

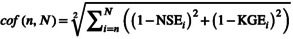

which represents the Euclidean distance to the optimal NSE and KGE = 1 over different soil depths (from *n* the uppermost soil layer to *N* the deepest). All sites were equally weighted to derive average final parameter sets for the subsequent spatial application. The 30 runs (from 5000) with the best performance over the entire soil profile (*cof*[1,5]) were selected and declared ‘behavioural’ runs, while the remaining runs were ‘non‐behavioural’. The two‐sample Kolmogorov–Smirnov test was used to test for parameter differences between behavioural and non‐behavioural runs. The maximum distance between the empirical cumulative distribution functions expressed by the behavioural and non‐behavioural parameter sets (test‐statistic *D*_max_) served as a measure to identify sensitive parameters (Harlin & Kung, 1992). Parameters were sampled from uniform prior distributions with boundary values derived from the literature (Federer, 2002; Federer et al., 2003; Groh & Puhlmann, 2013; Schmidt‐Walter et al., 2020) or, for the soil hydraulic parameters, within ±30% of the predicted value from the PTF. For the sensitivity analysis, 5000 parameter sets were sampled and classified into behavioural and non‐behavioural. The parameters with *D*_max_ values >0.2 in any of the main tree species plots were subsequently used as fitting parameters in the calibration procedure. These parameters were sampled 5000 times for model calibration, and the 30 best runs for each site (all over NSE 0.25) were selected for the forward model prediction. The calibration and validation procedure were done with the PTF of Wessolek et al. (2009) and Puhlmann and von Wilpert (2011). We used slope, aspect, latitude, species, and tree height as fixed parameters derived from site surveys.

For model validation, the transferability in time was tested by a temporal data split where the years 2015–2017 were selected for model calibration and the drought situation 2018 for model validation. The spatial transferability of the model was tested for 11 sites that were not included in the model calibration of the remaining 33 sites. From the 30 best runs at the 33 calibration sites, we derived median parameter sets for coniferous and deciduous forests to predict soil matric potential at the validation sites. These validation sites were selected to represent different regions and species. For spatial model application, median parameter sets for coniferous and deciduous forests of all 44 sites and calibrated to the period 2015–2018 were used. Model validation further involved comparing simulated throughfall amounts with those observed at 11 Swiss LWF sites (Thimonier et al., 2019). Finally, simulated actual evapotranspiration and soil evaporation were compared to eddy covariance measurements on one site with coniferous trees and another with predominantly deciduous trees. Turbulent fluxes of water vapour were measured continuously above the canopy using the eddy covariance method in two forest sites in Davos (coniferous) and Lägeren (mixed deciduous). In the coniferous forest in Davos, measurements were collected at 35 m and in the mixed deciduous forest in Lägeren at 47 m. Half‐hourly evapotranspiration fluxes were calculated using the EddyPro software (v6.1.0, LI‐COR Inc.). For more technical details about the eddy covariance measurements, see Paul‐Limoges et al. (2020) and Etzold et al. (2011).

### 2.5 SVAT‐based drought indices and early wilting

The model's ability to predict physiological drought was tested by intersecting simulated drought indices and soil matric potential with a Sentinel‐II based early‐wilting map of 2018 (Brun et al., 2020). As drought indices, we used the soil matric potential, deficit of plant‐available water in the root zone (from the mineral soil surface to the maximum rooting depth) (ADEF), the ratio of actual to potential transpiration (*T*_a_/*T*_p_), and relative plant‐available soil water in layers with roots (RELAWAT). In addition, we used a one‐sided, equal variance *t*‐test to assess differences in drought indices for pixels with and without early wilting.

GAMs (Hastie & Tibshirani, 1986) were used to test the ability of LWF‐Brook90‐derived drought indices to predict physiological drought represented by the presence of 2018 early wilting. The fraction of early wilting mapped on 10 m resolution of the Sentinel‐2 images within a 100 m resolution pixel (Brun et al., 2020) was used as the target variable. The explained deviance was used to assess the calibration strength of uni‐ and multivariate GAMs fitted 100 times to stratified random samples of 10,000 raster cells. A binomial error distribution was used. Response curves were derived from these univariate fits. As explanatory variables, in addition to the LWF‐Brook90‐derived drought indices, the set of predictors described in Brun et al. (2020) was used as explanatory variables (including variables describing vegetation structure, topography, soil properties, temperature, and precipitation). The model formulas were kept fixed but the degree of freedom for each smoother was optimized, except for categorical variables: such semi‐quantitative indices were modelled with smoothers of three degrees of freedom at maximum (Brun et al., 2020). The R package *mgcv* (Wood, 2006) was used to fit the GAMs. Research data are publicly archived in EnviDat (Meusburger et al., 2022).

## 3 RESULTS

### 3.1 SVAT model parameters and performance

Soil matric potential measurements in 44 soil profiles along the Swiss drought gradient from 2014 to 2018 successfully constrained the SVAT model. Parameter sensitivity measured by *D*_max_ identified the parameters with the strongest potential to optimize the combined objective function. *D*_max_ thereby depended on the predominant tree species and the two applied PTFs (Table S1).

For the PTF of Wessolek et al. (2009), the most sensitive parameter was the critical leaf water potential at which stomata are fully closed (psicr, MPa), followed by the shape of the root distribution (betaroot, unitless) and the maximum root depth (maxrootdepth, m). Of further importance were parameters that control potential transpiration, for example, the solar radiation level at which leaf conductance is half of its maximum, solar shortwave radiation at leaf level (r5, W m^−2^), maximum internal conductivity for water flow through the plants (mxkpl, day^−1^ MPa^−1^), and maximum leaf vapour conductance when stomata are fully open (glmax, m s^−1^). To a lesser extent, parameters controlling preferential soil water flow (ilayer, as an integer of the soil layer and infexp as the fraction of infiltration to the uppermost soil layer), the lower boundary condition (a drain value of 1 corresponding to free drainage), maximum leaf area index (maxlai, m^2^ m^−2^), and soil evaporation resistance at field capacity (rssa, s m^−1^). The saturated hydraulic conductivity of the soil (ksat, mm day^−1^) did not differ significantly between the best and remaining runs. For the PTF of Puhlmann and von Wilpert (2011), parameters controlling root distribution were most important, followed by the flow parameter ilayer and psicr. At the lower boundary of the soil profile, the soil hydraulic parameter ths (soil layer 5) and ksat (soil layer 4, 5) were sensitive. Furthermore, the fraction of internal aboveground plant resistance (the remaining resistance is attributed to the belowground root resistance) to water flow in the xylem (fxylem, 0–1) and the parameter controlling potential transpiration by setting VPD at which leaf conductance is halved (cvpd, kPa) were sensitive.

For the time‐split calibration period (2015–2017), the simulations with the PTF of Wessolek et al. (2009) yielded median NSEs of 0.52, 0.55, 0.37, and 0.26 for the average measurement depths of 20, 80, 110–150, and 140–200 cm, respectively. The KGE values were generally higher, with median values between 0.68 and 0.5 along the soil profile. For the PTF of Puhlmann and von Wilpert (2011), both performance criteria were lower, particularly for the deeper measurement depths (Table S2). Therefore, all the following results are based on the PTF of Wessolek et al. (2009). All NSE and KGE values originated from runs with the best performance for all measurement depths (minimum combined objective function *cof*[1,4]). Selecting the 30 best runs based on a single measurement depth (*cof*[1,1], *cof*[2,2], …) generally resulted in NSE >0.63 values for all 44 sites and depths.

The model's transferability in time and its capability to predict droughts were tested for the 2018 drought event. The temporal validation yielded better performance criteria than the calibration for both PTFs (Table S2). In contrast, the model's transferability in space to non‐calibrated sites using the median posterior parameters for deciduous and coniferous forests derived from the 33 calibration sites yielded with NSE_20_ = 0.29, NSE_80_ = 0.41, NSE_140_ = −8.51, NSE_190_ = −9.29 lower model performance, particularly for the deeper soil layers where for 8 of 17 sites the simulated conditions were wetter than measured (Table S2).

**FIGURE 2:**
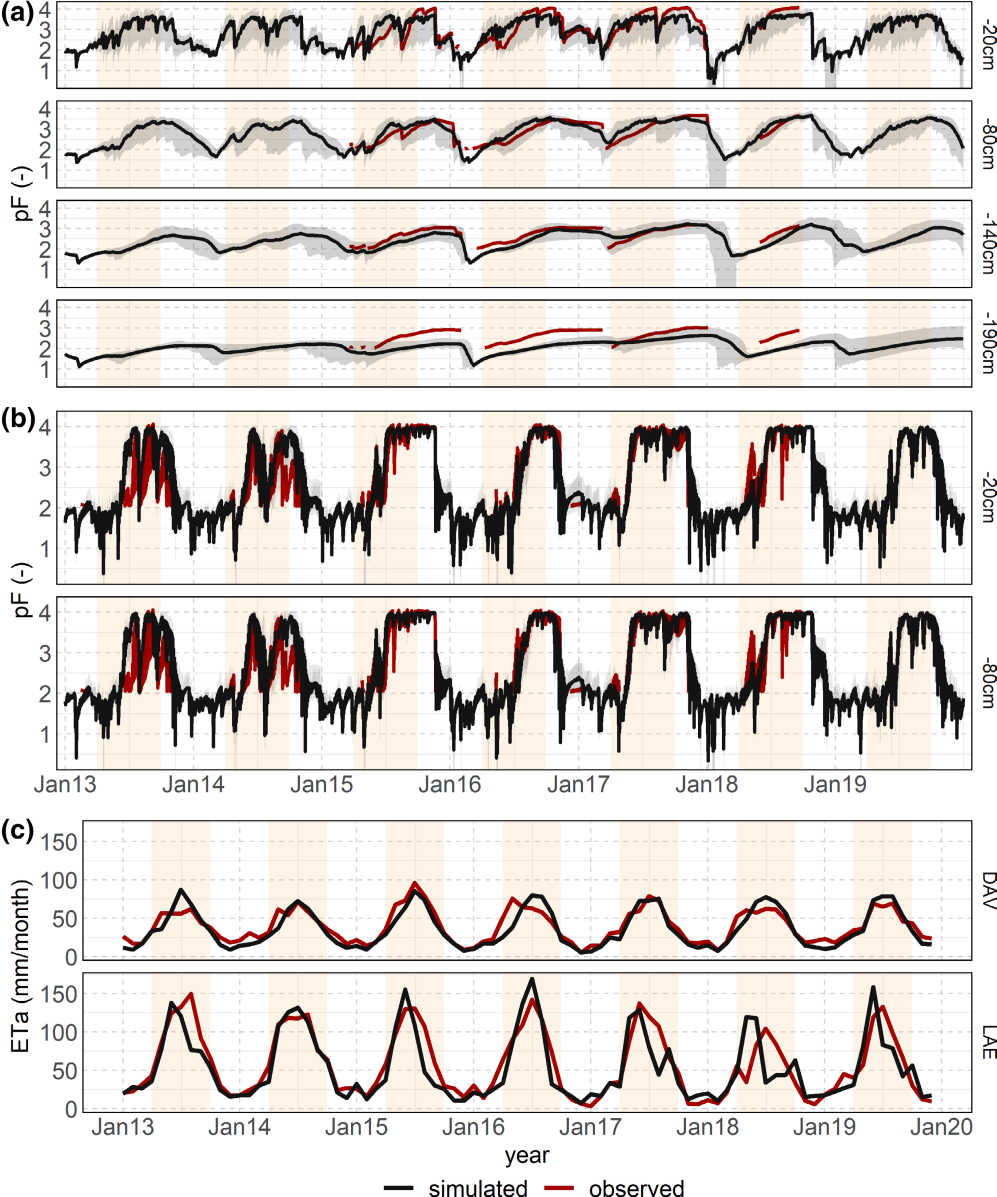
Simulated (black) and observed (red) pF (negative decadic logarithm of the matric potential in hPa) values in different soil depths (right *y*‐scale) exemplarily shown for sites with lower (a) and higher (b) model performance. Grey ribbons indicate the uncertainty resulting from parameter estimates. Summer half year from 1 April to 30 September in yellow. (c) Monthly eddy flux measured (red) and simulated (black) ETa fluxes for the two uncalibrated sites, Davos (DAV, coniferous) and Lägeren (LAE, deciduous).

Exemplarily, a site at the upper and lower end of model calibration performance is shown in Figure 2. A site with low overall model performance (NSE_20_ = 0.15, NSE_80_ = 0.58, NSE_140_ = 0.30, NSE_190_ = 0.03) was near Hohtenn in the Valais, with spruce on silty loam soil at 1140 m asl. One of the best fits (NSE_20_ = 0.75, NSE_80_ = 0.87) could be achieved at a site near Neunkirch in the Jura region, a beech site on a loamy and stony soil at 472 m asl. Both soils show drying in summer to pF values >4. However, we have to point out that values below pF 2 cannot be measured with the MPS‐2 sensor. The simulated onset of the transpiration matched well with the measurements, even though simulated budburst day of the year (DOY) was not included as a fitting parameter.

**FIGURE 3:**
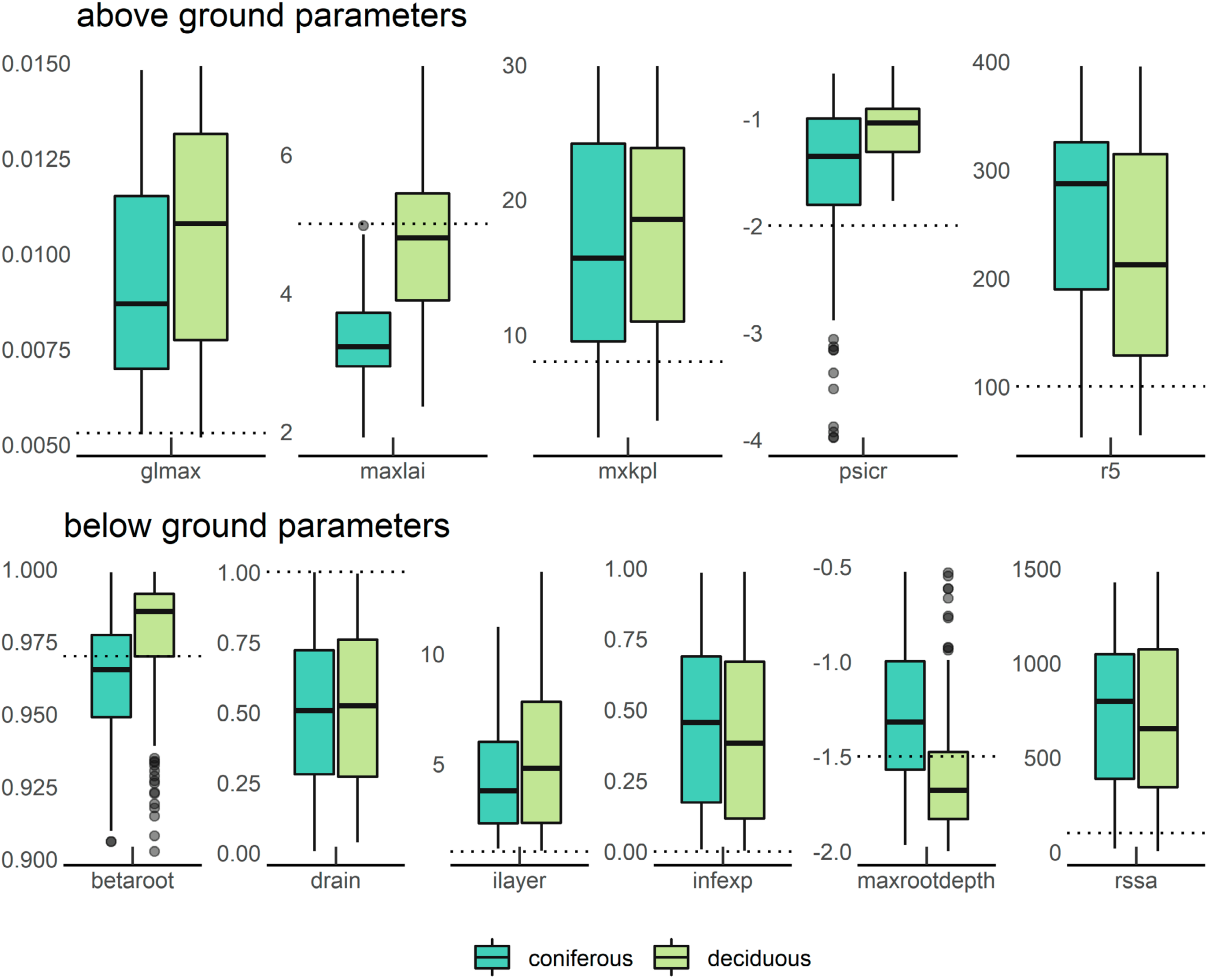
Posterior parameters estimated from best fits to measure matric potential data at 21 coniferous and 23 deciduous sites. LWF‐Brook90R default parameters are indicated as dotted horizontal lines. Aboveground parameters involve the maximum leaf vapour conductance when stomata are fully open (glmax, m s^−1^), the maximum leaf area index (maxlai, m^2^ m^−2^), the maximum internal conductivity for water flow through the plants (mxkpl, day^−1^ MPa^−1^), critical leaf water potential at which stomates fully closes (psicr, MPa), and solar radiation level at which leaf conductance is half of its maximum solar shortwave radiation value at leaf level (r5, W m^−2^). Belowground parameters involve the shape of the root distribution (betaroot, unitless), the lower boundary condition (a drain value of 1 corresponding to free drainage), preferential flow (ilayer, as an integer of the soil layer and infexp as the fraction of infiltration to uppermost soil layer), the maximum root depth (maxrootdepth, m), and soil evaporation resistance at field capacity (rssa, s m^−1^).

The median posterior parameters of behavioural runs were extracted for each dominant species (Table S3) as well as for deciduous and coniferous sites (Figure 3), subsequently used for the spatial model application. The fitted aboveground parameters were higher than the defaults for maximum leaf vapour conductance (glmax), maximum internal conductivity for water flow through the plants (mxkpl), and solar radiation level at which leaf conductance is half (r5). Less negative values compared to the default were obtained for the critical leaf water potential at which stomata close (psicr). The average values of measured maximum LAI were 4.6 for deciduous and 3.4 for coniferous stands. The corresponding fitted maximum LAI values were only 1% higher and 3% lower for deciduous and coniferous trees, respectively. Belowground fitted values showed with values of 0.5 that free drainage is hampered (a value of 1.0 would indicate free drainage, and 0.0 a no‐flux boundary), and values of ilayer and infexp above 0 indicate a macro‐pore‐assisted infiltration to deeper soil layers. Soil evaporation resistance at field capacity (rssa) was higher than the default value. The two parameters describing the root distribution differed between coniferous and deciduous sites with greater fitted maximum rooting depth (maxrootdepth) for deciduous trees and a smaller decline (higher betaroot) with soil depth as compared to coniferous sites (Figure 3, right). The mean maximum rooting depth of the studied easily rootable soils was fitted to 1.59 and 1.30 m for deciduous and coniferous trees, respectively.

Comparing simulated evapotranspiration fluxes for the two uncalibrated sites using the median parameter sets with eddy covariance measurements of ET_a_ above‐canopy (Davos and Lägeren) indicates a good agreement regarding the seasonal course and deviations of absolute fluxes (Figure 2c). In winter, ET_a_ was underestimated for Davos while it was overestimated for Lägeren. At Lägeren, the simulated ET_a_ peaks in July were higher than observed except for 2017, and the maximum daily ET_a_ of 8.0 mm was observed on 25 July 2015, and simulated at 5.9 mm. Lateral water fluxes along the slope might cause higher discrepancies in Lägeren. While LWF‐Brook90 does have a downslope module, it does underestimate incoming water from uphill at sites with steep slopes like Lägeren and therefore tends to underestimate water availability. The maximum daily simulated surface runoff and drainage fluxes in early January 2018 are 69.6 and 38.3 mm, several times higher than the fluxes returning to the atmosphere.

At the 11 Swiss LWF sites, uncalibrated simulated interception evaporation (*E*_i_, as the difference between bulk precipitation and throughfall) was simulated with a mean value of 14.8% ± SD 2.4%, which is in the range of measured *E*_i_ of 14.0% ± SD 7.8%. The observed variability of interception was higher than the modelled, with the highest interception losses at the *Picea abies* site near Vordemwald and the lowest at the *Pinus sylvestris* site near Visp, where LAI is low due to a high mortality rate. The variability between sites was smaller for simulated values.

### 3.2 Storage capacity of plant‐available soil water in Swiss forests

**FIGURE 4:**
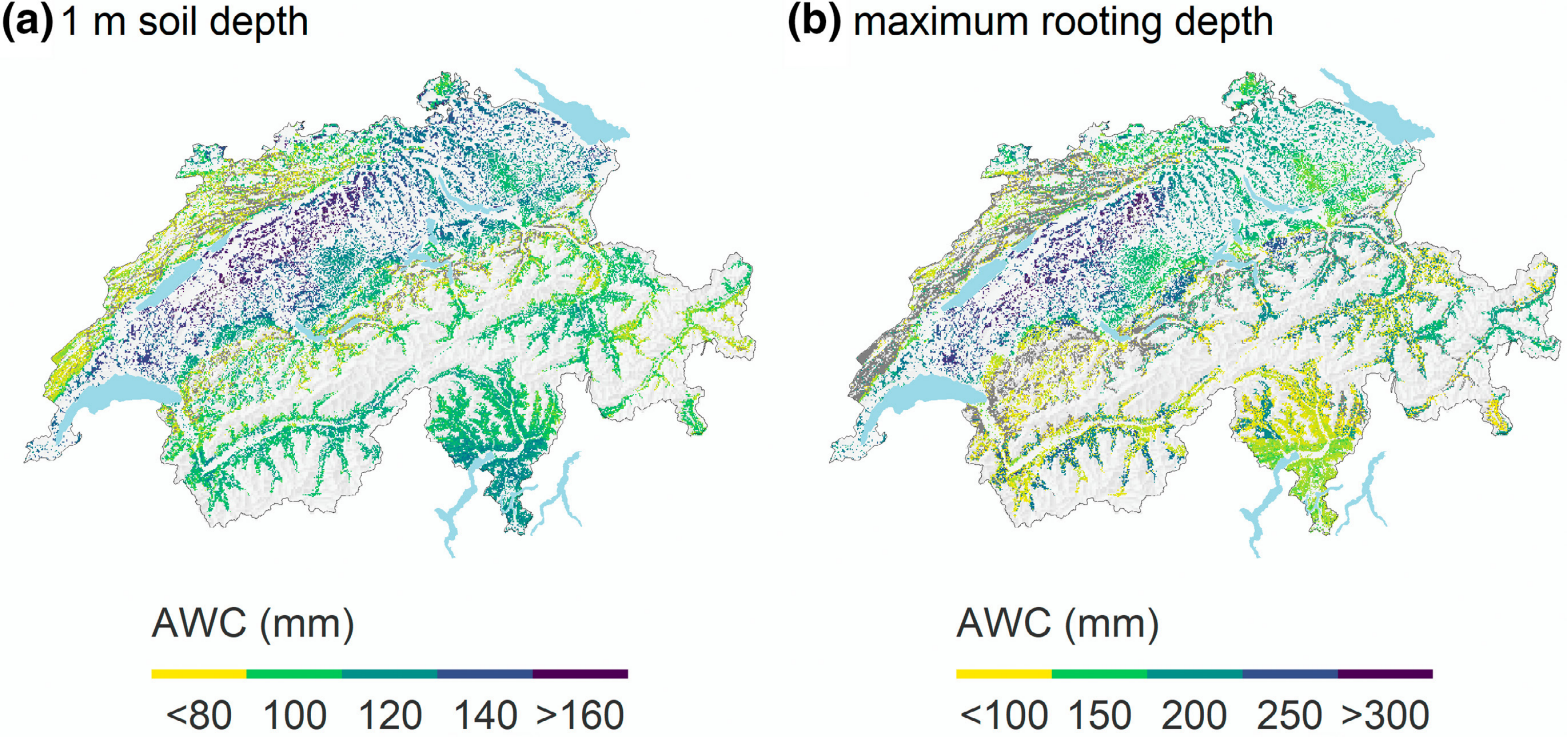
Available water storage capacity (AWC in mm) of Swiss forest soils until (a) 1 m soil depth and (b) the mrd. Estimates are calculated according to the pedotransfer function of Wessolek et al. (2009) applied to regionalized, layered soil properties and maximum potential root depth information (Baltensweiler et al., 2021). Grey pixels represent the hill shade of non‐forested areas.

The layered spatial soil property maps combined with the PTF‐derived soil hydraulic parameters allowed us to predict the plant‐available soil water storage capacity (AWC) for the forested area of Switzerland. The resulting median AWC that Swiss forest soils retain against gravitation amounted to 137 ± 42 mm when integrated from the surface to the regionalized mrd with a median depth of 1.19 m. Across Switzerland, values ranged from 53 mm in mountain areas to 341 mm on the Swiss Plateau (Figure 4). The total AWC volume summed to 2.0 ± 0.6 km^3^ above mrd. The median AWC decreased to 107 ± 12 mm and a storage volume of 1.44 ± 0.16 km^3^ if 1 m soil depth was assumed as the lower boundary for roots. Under saturated conditions, additional gravitational water (GWC) of 71 ± 40 mm (until 1 m) and 91 ± 58 mm (until mrd) was estimated (Figure S2). The uncertainties for these water pools accumulate when summed over the six different soil depths (Figure S3). The average coefficient of variation (CV) for the six soil layers ranges between 0.13 and 0.18 for AWC and GWC. The mrd introduces the most considerable uncertainty to AWC, with a mean CV of 0.42. Across Switzerland, neither the AWC above 1 m nor the AWC above mrd differed between predominantly deciduous and coniferous stands. The fractions of average AWC and GWC per volume of soil decrease with soil depth (Figure S4), resulting in an AWC that is 36% higher between 0 and 1 m than between 1 and 2 m soil depth.

### 3.3 Swiss forest water fluxes of drought and non‐drought years

**FIGURE 5:**
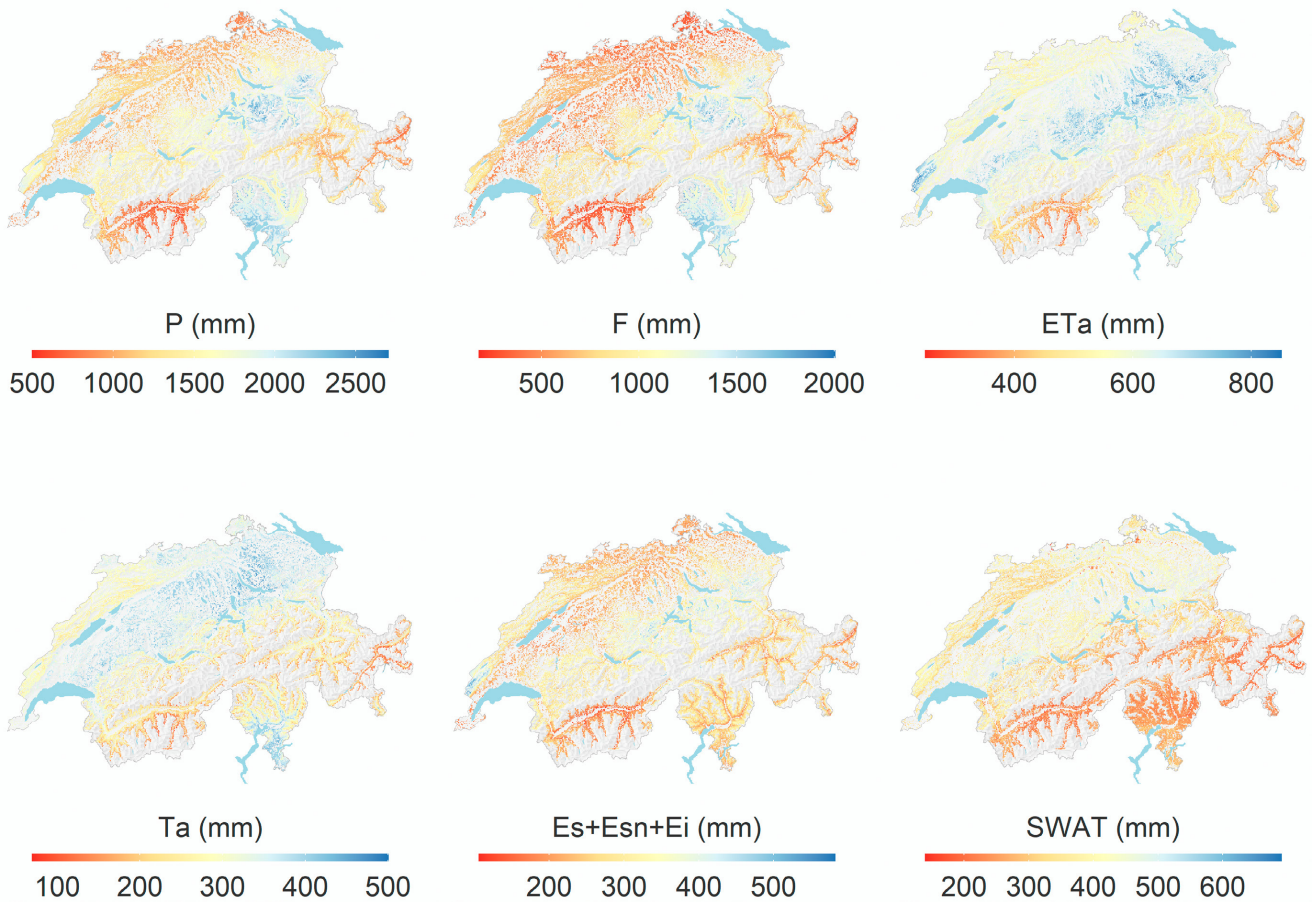
Yearly average (2014–2019) of precipitation (*P*), actual evapotranspiration (ET_a_), evaporation as the sum of soil, snow and interception evaporation (*E*), actual transpiration (*T*_a_), runoff (*F*), and total soil water storage (SWAT).

Water fluxes across Switzerland for non‐drought years (Table 1) showed a mean annual actual evapotranspiration (ET_a_) flux of 571 mm, with the maximum rate of 888 mm observed in the Northern Alps and the minimum of 243 mm in a dry inner alpine valleys (at 1730 m asl) with shallow mrd (Figure 5). Actual transpiration (*T*_a_) accounted on average for 52% of the ET_a_ flux, with higher shares in the Swiss Plateau and the Ticino. Runoff (*F*) showed the highest rates in the eastern part of the Northern Alps and the Ticino. Total soil water in all layers at the end of each year (total soil water in all layers = SWAT) was on average 327 mm and generally higher on the Swiss Plateau than in the Alps and Ticino. In the studied period, the share of precipitation returning to the atmosphere by ET_a_ was 41%, and the runoff fraction was 57%. The annual average transpiration deficit (*T*_d_) was estimated as 42 mm and thus much lower than the maximum values simulated at site level in the lower altitudes of Valais with up to 350 mm. Deciduous and coniferous species separated precipitation slightly differently between ET_a_ and *F*. While *T*_a_ was quite similar for the deciduous and coniferous trees, they differed in the higher generation of runoff by sites with deciduous trees, which mainly occurred due to lower interception losses during winter. In drought years (Table 1), absolute fluxes of *F* and the sum of evaporation fluxes (*E*) were smaller. However, the fraction of *F* in relation to precipitation was higher with 58% and 60% for the 2015 and 2018 droughts, respectively. The most distinct difference in drought years was the increased *T*_d_. Trees reduced the potential transpiration by 23% (in 2015) and 28% (in 2018), compared to 12% in non‐drought years.

**TABLE 1:** Simulated annual water fluxes (mm) per drought, non‐drought years, and deciduous (dec) and coniferous (con) trees. Precipitation (*P*), runoff (*F*), evapotranspiration (ET_a_), actual transpiration (*T*_a_), potential transpiration (*T*_p_), transpiration deficit (*T*_d_ = *T*_p_ − *T*_a_), evaporation of soil (*E*_s_), evaporation of snow (*E*_sn_), interception on leaves (*E*_i_), and the sum of evaporation fluxes (*E*)

Year	Tree	*P*	*F*	ET_a_	*T*_a_	*T*_p_	*T*_d_	*E*_s_	*E*_sn_	*E*_i_	*E*
2015	con	1182	674	547	283	379	96	89	63	111	264
2015	dec	1135	677	508	289	368	79	98	46	74	219
2018	con	1230	727	554	294	415	121	82	67	112	261
2018	dec	1190	724	511	293	308	105	94	46	78	218
Non‐drought	con	1385	761	594	294	340	46	106	64	131	300
Non‐drought	dec	1364	797	550	299	337	38	117	44	90	251

**FIGURE 6:**
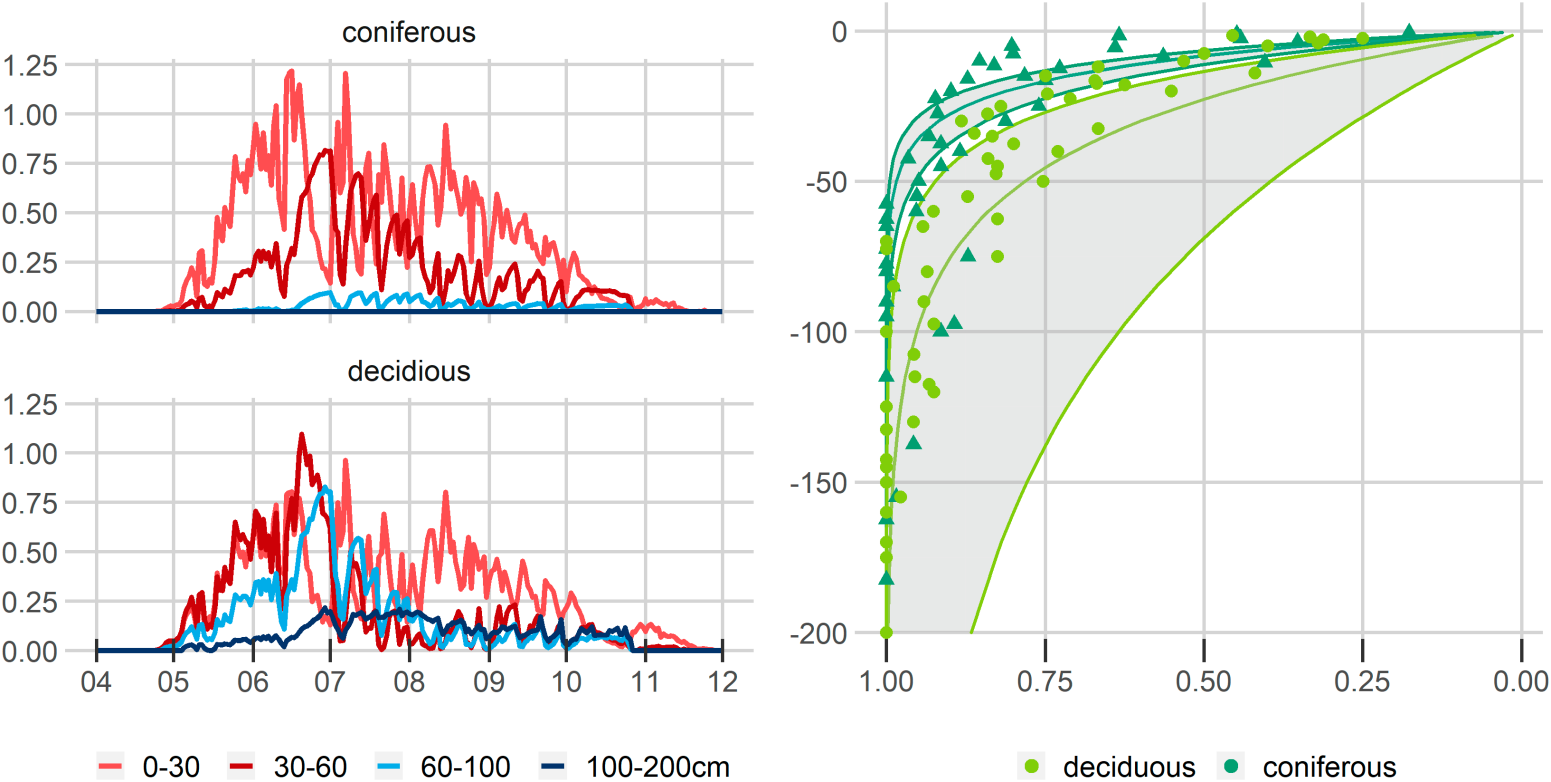
Simulated root water uptake aggregated for different soil depths per deciduous and coniferous trees across Switzerland at daily resolution (left panel). Cumulative root fraction per soil depth for coniferous and deciduous sites (points) and posterior depth distribution parameter, beta (lines, right panel).

On a Swiss scale, monthly water uptake from deeper soil layers is larger for deciduous trees, with the largest share simulated to originate from 0 to 60 cm soil depth, while for the coniferous trees, the maximum water share was derived from the 0 to 30 cm soil layer (Figure 6, left panel). However, daily transpiration per soil layer shows a clear shift in plant water sources in July 2018, where after the drying out of the upper layers, a larger share of water uptake by roots was simulated for deeper layers. For deciduous trees, this shift to deeper water sources was more pronounced and persistent (Figure 6, left panel). After drying of the deep layers at deciduous sites, the uppermost soil layer became the primary water source for the remainder of the year due to rain events. The difference in soil water uptake resulted from the different root distribution of coniferous and deciduous trees (Figure 6, right panel).

### 3.4 Drought indices and thresholds for 2018 early wilting

**FIGURE 7:**
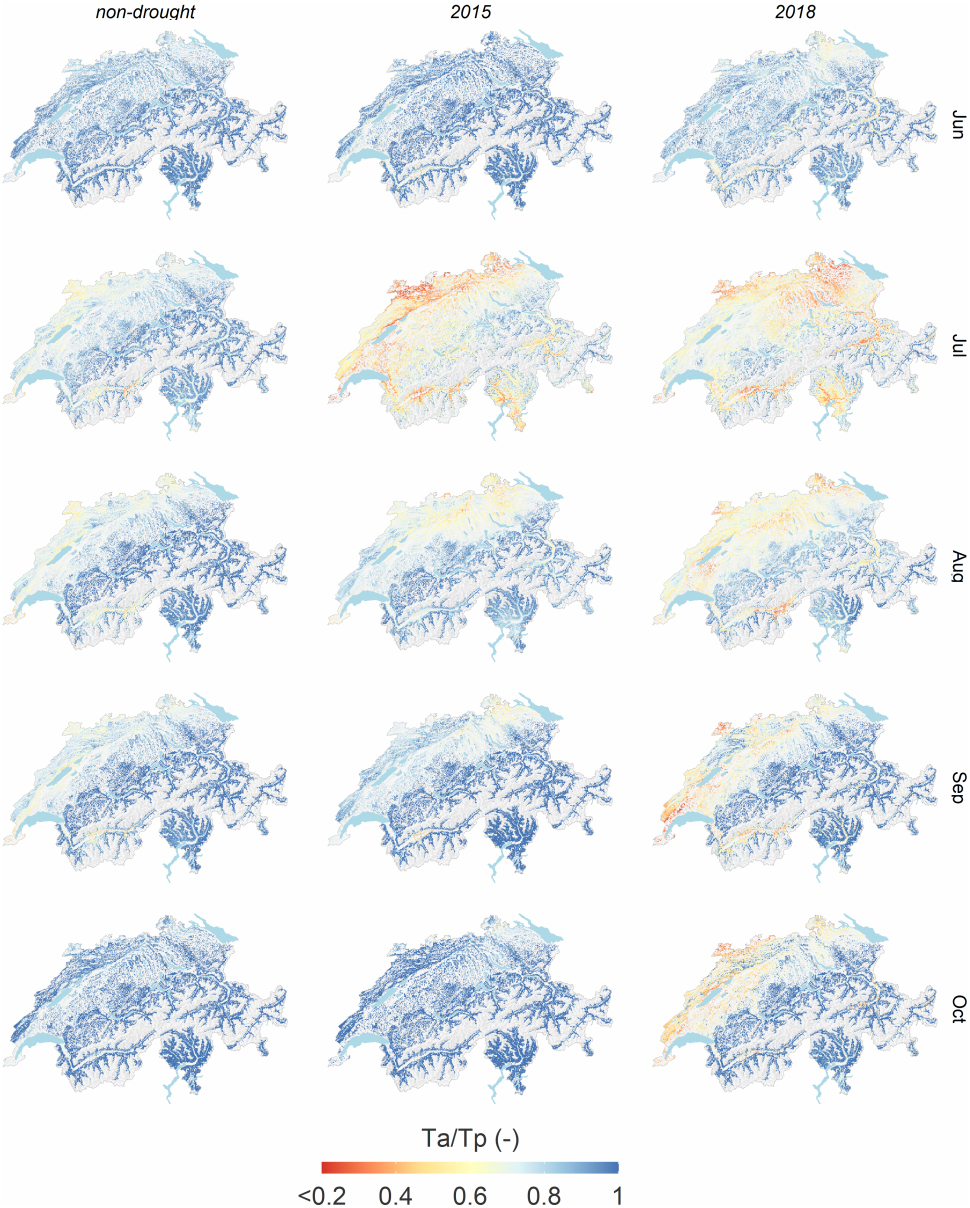
Ratio of actual to potential transpiration (−) as mean of non‐drought years 2014, 2016, 2017, 2019 (left) and 2015 (middle) and 2018 (right) for the month June, July, August, September, and October (rows). The smaller the ratio, the larger the predicted reduction of actual transpiration due to soil drought.

The ratio of actual to potential transpiration (*T*_a_/*T*_p_) as a measure of plant water supply to atmospheric demand shows distinctly lower ratios in 2015 and 2018 than in non‐drought years (Figure 7). In the summer of 2015, low ratios occurred instantaneously across large forest areas of Switzerland, with hotspots in the northern Jura mountains and low‐elevation areas of Valais and Ticino. Low ratios persisted until August and September in the eastern part of the Swiss Plateau. In 2018, the *T*_a_/*T*_p_ was already relatively small in June. In July, the hotspots occurred in the northeastern Swiss Plateau, at a lower elevation of Valais and Ticino and the Grison area. In September, the north‐western part of Switzerland and the region north of Lake Geneva were still dry. In several regions, the 2018 drought persisted longer than the 2015 drought, and low ratios occurred still in October. The higher intensity predicted for the 2018 drought can also be expressed by the average *T*_d_, which was with 18 mm (June), 48 mm (July), 29 mm (August), and 12 mm (September) higher in 2018 than with 7 mm (June), 53 mm (July), 21 mm (August), and 5 mm (September) in 2015.

**FIGURE 8:**
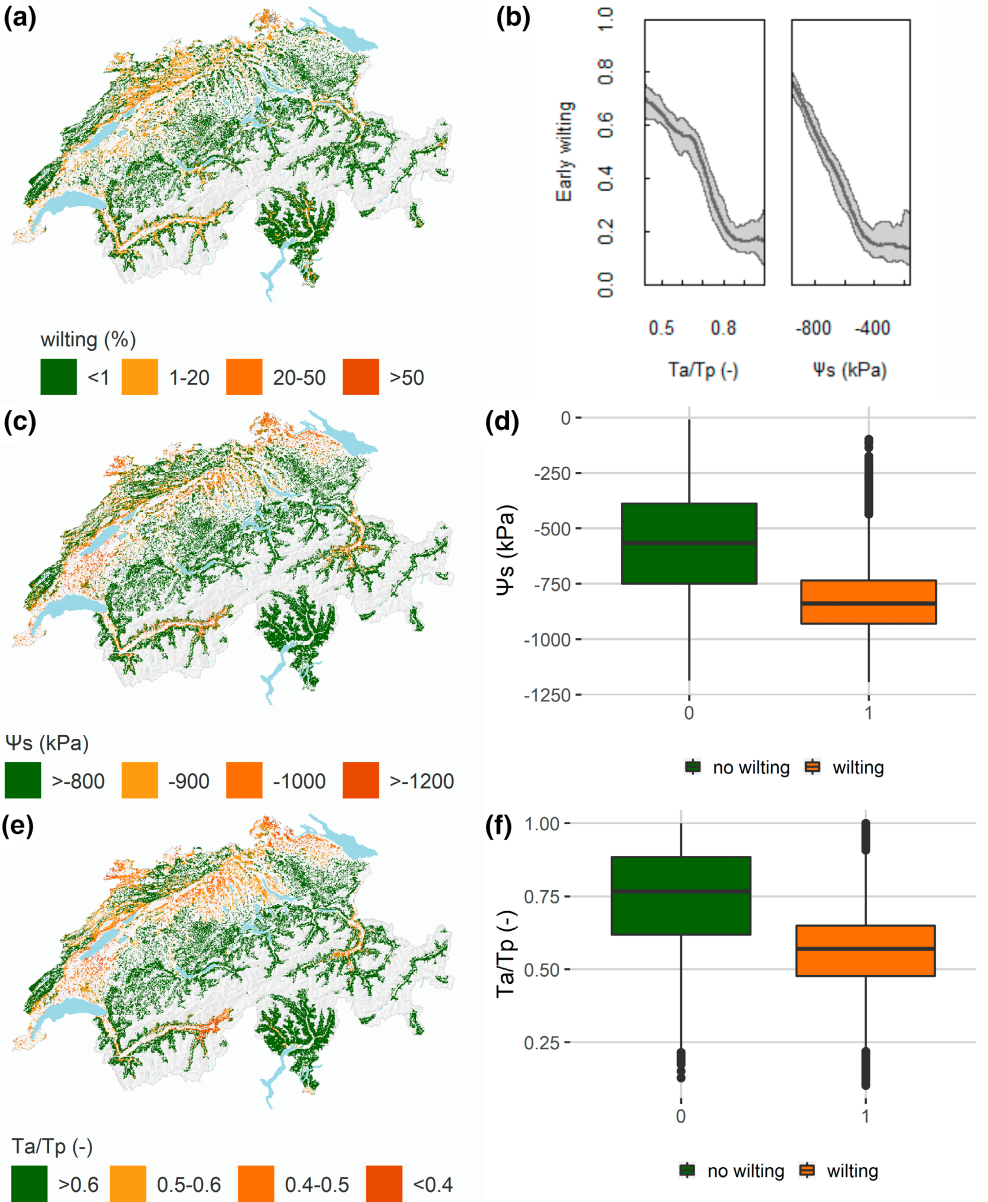
Comparison of (a) early‐wilting occurrence (percentage occurrence within a 500 m pixel) in August 2018 (adapted after Brun et al., 2020) and (b) univariate response curves of mean soil matric potential in the rooting zone (*ψ*_s_) and actual to potential transpiration *T*_a_/*T*_p_. (c) Map of mean soil matric potential in the rooting zone in August 2018 and (d) boxplot of soil matric potential in pixels with and without early wilting. (e) Map of actual to potential transpiration (*T*_a_/*T*_p_) in August 2018 and (f) boxplot of *T*_a_/*T*_p_ in pixels with and without early wilting.

Across Switzerland, modelled drought indices in 2018 matched well with a remotely sensed early‐wilting map, for which they were excellent predictors. Mean soil matric potential in the potential rooting zone in August 2018 showed with 20% univariate explained deviance higher explanatory power than the best climatic predictor, despite the coarser spatial resolution. The univariate explained deviance fell to 15% and 12% when only the upper 60 cm and 30 cm of the mean soil matric potential were considered. Low soil matric potential matched the early‐wilting hotspot close to Lake Constance and the north‐western part of Switzerland, while the match was worse in the Jura mountains and in Grisons (Figure 8). The drought stress index *T*_a_/*T*_p_ had a univariate explained deviance of 16%. Areas with low *T*_a_/*T*_p_ matched the hotspot regions of early wilting, except the Jura mountains and the Ticino. These missing regions were captured by the minimum daily *T*_a_/*T*_p_ values simulated in August. Other drought indices such as ADEF and RELAWAT showed lower explained deviances of 3% and 11%, respectively.

The mean *T*_a_/*T*_p_ significantly differs (*p* < .001) between pixels with (*T*_a_/*T*_p_ = 0.57) and without early wilting (median *T*_a_/*T*_p_ = 0.75). The same (*p* < .001) is observed for soil matric potential, where the median soil matric potential in the rooting zone of browning affected pixels is −818 kPa and without browning, −562 kPa.

## 4 DISCUSSION

### 4.1 Model performance and posterior parameters

For SVAT models, a major source of uncertainty results from parameter estimates and equifinality due to the higher data demand than data availability (Beven & Kirkby, 1979). The sensitivity analysis helped identify parameters that significantly affect the model performance. The model sensitivity to a given parameter thereby depended on the model structure, the objective function, and target variables within the objective function. The most sensitive parameters for the measured soil matric potential under predominantly dry conditions were the parameters controlling stomatal closure (psicr) and root distribution, followed by plant hydraulic conductivities. None of the soil hydraulic parameters was sensitive, even though the saturated hydraulic conductivity (Ksat) was previously reported as a highly sensitive parameter (Walthert et al., 2015). This is likely due to the limited measurement range of our sensors (*ψ*_s_ < −10 kPa), where non‐mobile water predominates, which was less suitable to constrain Ksat.

All soil matric potential measurements of a soil profile were combined in the objective function with the intention to simulate the water balance along the entire soil profile correctly. This procedure reduced the performance metric, but the achieved NSE and KGE values can be considered behavioural (Knoben et al., 2019). Fitting the model to a single observed depth enhanced the performance criteria but at the cost of unrealistic parameter estimates. For all sites, we observed a bias in the first year. The destruction of roots during sensor installation was likely responsible for this bias towards wetter observed conditions after installation. The temporal validation with the 2018 drought demonstrated the model's capability to predict future drought since the performance criteria improved compared to the calibration period. The spatial transferability of the model was less satisfactory as the agreement between observed and simulated soil matric potential decreased remarkably for the deeper soil layers (with wetter simulated conditions). Still, the magnitude of interception and ET_a_ at uncalibrated sites was predicted accurately.

Posterior parameters related to plant conductivity (glmax, mxkpl, and r5) were all higher than the default LWF‐Brook90 parameters. The higher fitted mean glmax values of 0.011 m s^−1^ (deciduous trees) and 0.0087 m s^−1^ (coniferous trees) are within reported ranges for *Fagus sylvatica* and *Quercus spp*. of 0.0002–0.018 m s^−1^ and *Picea abies* and *Pinus sylvestris* of 0.007–0.013 m s^−1^ (Bréda et al., 2006; Köstner, 2001; Schmidt, 2007). In contrast, the fitted psicr had median values of −1.4 MPa for deciduous and −1.1 MPa for coniferous trees, which was higher than the default settings indicating an earlier stomata closure. The fitted psicr values were at the higher spectrum of leaf water potential compared to minimum leaf water potential reported across Germany, Switzerland, and Austria (Arend et al., 2021, 2022; Walthert et al., 2021; Zweifel et al., 2007). These studies found minimum leaf water potential of *Fagus sylvatica* in the range of −1.2 to −3.3 MPa, of *Quercus spp*. from −1.8 to −4.0 MPa, and of *Pinus sylvestris* and *Picea abies* from −1.4 to −2.5 and − 0.9 to −4.2 MPa, respectively. However, most studies did not report the degree of stomatal closure related to these leaf water potentials. Belowground, the posterior parameters indicated the occurrence of preferential flow with approximately only 30%–60% of rainfall water infiltrating to the first layer (~upper 20 cm of the soil), while the remainder of water infiltrated to deeper soil layers. Furthermore, the posterior root parameters indicated that the roots of deciduous trees reached deeper and root density declined less rapid with soil depth than the roots of coniferous trees, which agrees with global observations of shallower rooting systems of evergreen needle‐leaf trees (Fan et al., 2017). For the posterior resistance to soil evaporation at field capacity, high values compared to LWF‐Brook90 default parameters were fitted, but the values were still not higher than found in a comparable study (Schmidt‐Walter et al., 2020). The omission of the organic layers might have been responsible for this, as organic layers may prevent soil evaporative losses. In general, the model was fitted to measurements drier than field capacity (*ψ*_s_ < −10 kPa). Thus, the applicability of the model parameters to wet, mobile soil water conditions still needs (*ψ*_s_ > −10 kPa) to be tested.

### 4.2 Plant‐available water storage capacity

AWC is the water pool held at a soil matric potential between field capacity and the wilting point (adjusted by gravel content) potentially available for plants during drought. AWC was shown to be an essential basis for modelling ecosystem water balance (Granier et al., 1999), tree growth (Guillemot et al., 2017), and survival (Preisler et al., 2019). The presented AWC map is based on the PTF of Wessolek et al. (2009), which produced overall the best calibration results. This PTF uses the German soil textural classes to predict the Mualem–van Genuchten parameters; bulk density and soil organic matter are not considered. The uncertainty of the AWC map was partly reduced by the discretization step of sand, silt, and clay fraction to textural classes. The mrd introduced the largest uncertainty. The presented assessment did not include the AWC of organic layers that can be considerable in forests and therefore underestimates the AWC presented here, particularly for coniferous sites at high elevation. An overestimation of AWC is expected in regions with anaerobic soil horizons that are less rootable such as in Flysch areas or depressions on the Swiss Plateau.

The presented AWC map is, to our knowledge, the first one to take advantage of regionalized and depth‐resolved soil properties. A previous AWC map (until 1 m soil depth) of Swiss forests soils was based on 1234 soil profiles grouped into AWC classes according to Eckelmann (2005) and regionalized by assigning them to lithological classes (Remund & Augustin, 2015).

### 4.3 Swiss forest water balance

The presented mean water balance (2014–2019) complies with the evaporation and transpiration fractions expected for Switzerland, such as a maximum ET_a_ of 800 mm year^−1^ (Schädler & Weingartner, 2002). The estimated ET_a_ was 5% and 7%, and *T*_a_ was 9% and 4% lower than simulated for coniferous and deciduous trees in Switzerland by Zierl (2001), and also 7% lower than eddy covariance‐derived measurements from two sites. Accordingly, the runoff proportions are with 57% higher than the identified 51% by Zierl (2001).

We hypothesized that a lower proportion of blue water (water that drains to the freshwater system) in dry years was driven by an increased ET_a_ flux, as predicted by Mastrotheodoros et al. (2020) for the entire Alpine mountain range. The first part of the hypothesis was confirmed (Table 1). On average, 12% to 8% less flow (runoff+drainage) occurred for deciduous and coniferous areas in both drought years. However, our findings suggest that this flow deficit in dry years was not amplified by higher ET_a_ fluxes during drought years in forests. Indeed, the drying out of the topsoil layers seems to have caused a lower *E*_s_ flux, and the precipitation deficit led to a lower *E*_i_ flux in dry years. The Swiss forests mean *T*_a_ showed only marginal differences between coniferous and deciduous trees and also between drought and non‐drought years. We explain this surprising stability in the *T*_a_ flux by the shift in RWU from the upper to the lower layers following the preceding drought and through averaging across sites that are energy limited where *T*_a_ is higher and water‐limited sites where *T*_a_ is lower during drought. By stomatal closure, trees saved 8% and 9% of the incoming yearly precipitation in 2015 and 2018. The high variability of *T*_d_ between drought and non‐drought years emphasizes trees' plasticity to adjust transpiration to varying climatic conditions.

Regarding tree species, coniferous trees, on average, were returning a larger share of precipitation via leaf interception and snow evaporation to the atmosphere than deciduous trees. The *T*_d_ is slightly higher for coniferous trees, even though the deciduous and coniferous areas' AWC (until mrd, 1 and 2 m) does not differ. The higher *T*_d_ for coniferous trees likely results from the shallower root distribution limiting water supply earlier. For coniferous trees, source water originates predominantly from the topsoil (approx. 30 cm) and only during a short time window deeper water becomes relevant. On the other hand, deciduous trees were simulated to take up water also from deeper layers, particularly in spring and summer. Topsoil water sources dominated after the summer of 2018 and the drying of deeper layers. This difference in source water depth between species and with successive drying agrees with the findings of Brinkmann et al. (2018), observing deeper water sources for beech than for spruce trees. However, it is expected that there is a large variability between the species merged here into deciduous or coniferous groups, for example, between beech and pubescent oak or even between trees of the same species growing under different edaphic conditions as reported in measurement studies, for example, for hydromorphic conditions (Gessler et al., 2022) or having a different acclimation legacy (Gao et al., 2021; Zweifel et al., 2020).

### 4.4 Physiological drought prediction capacity

Mean soil matric potential in the potential rooting zone was identified as the most powerful predictor for spatial patterns of 2018 early wilting in Switzerland. The better performance of our 500 m resolution outputs compared to 100 m resolution climatic variables underlines that the relevance of the information contained in the coarsely resolved soil water status predictors outcompetes the substantially higher density of information contained in the more finely resolved climatic predictors. As stressed by Piedallu et al. (2011) and hypothesized Sturm et al. (2022), it was shown that detailed 3D soil information improves physiological drought stress prediction. The smaller univariate explained deviance by soil matric potential in the upper 60 cm compared to soil matric potential in the entire rooting zone, and the switch to deeper source water during dry topsoil conditions indicates that deeper soil water is a critical source during drought conditions buffering the impacts of dry climatic conditions. Areas with early wilting showed a significantly lower soil matric potential with mean values close to the recently estimated threshold of −800 kPa for mature beech trees (Walthert et al., 2021). Here, we want to point out that we merged species with likely different wilting thresholds.

The drought index *T*_a_/*T*_p_ performed almost as well as soil matric potential in predicting 2018 early wilting. The *T*_a_/*T*_p_ spatiotemporal pattern highlighted the longer persistence of the 2018 drought in autumn than the 2015 drought. Furthermore, the duration and the drought intensity were higher in 2018 than in 2015, as indicated by higher cumulative *T*_d_ over the summer months. All this might be responsible for further visible crown damages after 2018 (Braun et al., 2020; Brun et al., 2020; Sturm et al., 2022) but not after 2015.

## 5 CONCLUSIONS

The interlinkages between aboveground and belowground via a mechanistic SVAT model revealed that Swiss forests are strongly feedback driven under drought. Forest trees reduced the potential transpiration by 23% (in 2015) and 28% (in 2018). Furthermore, modelled evaporative fluxes were smaller in dry years due to (i) the precipitation deficit causing lower interception loss and (ii) lower soil moisture causing less soil evaporation. Thus, for the Swiss forest area, our findings do not confirm the amplification of the 2015 and 2018 drought by an enhanced ET_a_ flux as predicted for the Alpine mountain range. In contrast, the lower evaporation flux and the higher *T*_d_ counteract the water deficit, emphasizing the strong regulatory capacity of the studied forest ecosystem.

In 2018, large‐scale early wilting was observed in Switzerland, and the *T*_d_ confirmed a longer duration and intensity of the 2018 drought than in 2015. Under the highest Representative Concentration Pathway 8.5, the risk of such droughts is expected to increase by seven times until the second half of the century affecting large parts of Central Europe (Hari et al., 2020). The simulated soil matric potential and the drought index *T*_a_/*T*_p_ were significant predictors of 2018 widespread early wilting in Switzerland, despite the substantially lower information content than spatially higher resolved climatic variables. Increasing the spatial resolution of the SVAT model would likely further improve its predictive capacity of drought‐induced early wilting. Furthermore, with its low computation demands and its iterative coupling to remote‐sensing‐derived forest characteristics, the model has a high potential to be transformed into an operational nowcasting tool.

Our study emphasized the importance of belowground processes. The sensitivity of the root parameters and the buffering effect of deeper soil water during dry periods stress the importance of these processes for physiological drought prediction. Still, the actual root distribution and RWU by individual species and their modulation by edaphic, climatic, and acclimation processes remain a major uncertainty and opportunity for improvement in predicting physiological drought.

## CONFLICT OF INTEREST

All authors declare no conflict of interests.

## Supporting information


Table S1
Table S2
Table S3
Figure S1
Figure S2
Figure S3

## Data Availability

The data that support the findings of this study are openly available in EnviDat at: 10.16904/envidat.335
