# Supplementary material for: Soil–plant interactions modulated water availability of Swiss forests during the 2015 and 2018 droughts
Source: Glob Chang Biol. 2022 Jul 24;28(20):5928–44. doi: 10.1111/gcb.16332 (PMC9546155; doi:10.1111/gcb.16332)
Supplement: Supplementary file 1 — Table S1 Table S2 Table S3 Figure S1 Figure S2 Figure S3 [file GCB-28-5928-s001.docx]

**Soil-plant interactions modulated water availability of Swiss forests during the 2015 and 2018 droughts**

Katrin Meusburger*^1^, Volodymyr Trotsiuk^1^, Paul Schmidt-Walter^2^, Andri Baltensweiler^1^, Philipp Brun^1^, Fabian Bernhard^1^, Mana Gharun^3,4^, Raphael Habel^5^, Frank Hagedorn^1^, Roger Köchli^1^, Achilleas Psomas^1^, Heike Puhlmann^5^, Anne Thimonier^1^, Peter Waldner^1^, Stephan Zimmermann^1^, Lorenz Walthert^1^

**Contact** **information**: [katrin.meusburger@wsl.ch](mailto:katrin.meusburger@wsl.ch), +41 44 739 2490

1. Swiss Federal Institute for Forest, Snow and Landscape Research (WSL), CH-8903 Birmensdorf, Switzerland.
2. Agrometeorological Research Center, German Weather Service (DWD), Bundesallee 33, 38116 Braunschweig, Germany.
3. Department of Environmental Systems Science, ETH Zürich, Universitätstr. 2, 8092 Zürich, Switzerland.
4. Department of Geosciences, University of Münster, GEO1, Heisenbergstrasse 2, D-48149 Münster, Germany.
5. Department of Soil and Environment, Forest Research Institute Baden Württemberg, Freiburg, Germany

# Supplementary Information

Supplementary Table 1 Maximum difference (D_max_) between the empirical cumulative distribution functions of parameter values resulting from high performance vs low-performance simulations regarding measured matric potential of the calibration period (2015-2017). D_max_ values are displayed per forest stand and pedotransfer function (PTF), with W referring to the PTF of Wessolek et al., 2009 and P to Puhlmann and Wilpert, 2011. D_max_ values exceeding 0.2 (= significant parameter after Harlin and Kung, 1992) were selected as fitting parameters.

| PTF | | parameter | description | beech | oak | beech/oak | spruce | pine | spruce/pine | D_max_ |
| --- | --- | --- | --- | --- | --- | --- | --- | --- | --- | --- |
| W | | alb | Albedo of soil/vegetation surface without snow (-). | 0.14 | 0.17 | 0.16 | 0.15 | 0.16 | 0.17 | 0.17 |
| W | | albsn | Albedo of soil/vegetation surface with snow (-). | 0.16 | 0.14 | 0.17 | 0.19 | 0.18 | 0.19 | 0.19 |
| W | | drain | Switch for lower boundary condition to be free drainage (1) or no flow (0). | 0.18 | 0.17 | 0.20 | 0.17 | 0.15 | 0.17 | **0.20** |
| W | | ilayer | Number of layers from top to which infiltration is distributed (-). | 0.22 | 0.20 | 0.20 | 0.20 | 0.23 | 0.21 | **0.23** |
| W | | infexp | Shape parameter for distribution of infiltration in first ILayer, for value 0 infiltration is in top layer only (-). | 0.18 | 0.18 | 0.16 | 0.20 | 0.18 | 0.17 | **0.20** |
| W | | bypar | Switch to allow (1) or prevent (0) bypass flow in deeper layers (-). | 0.16 | 0.16 | 0.18 | 0.17 | 0.15 | 0.12 | 0.18 |
| W | | glmax | Maximum leaf vapour conductance when stomata are fully open (m s^-1^). | 0.17 | 0.20 | 0.19 | 0.13 | 0.18 | 0.24 | **0.24** |
| W | | cvpd | Vapour pressure deficit at which leaf conductance is halved (kPa). | 0.16 | 0.13 | 0.19 | 0.16 | 0.18 | 0.18 | 0.19 |
| W | | r5 | Solar radiation level at which leaf conductance is half of its value at maximum shortwave radiation (W m^-2^). | 0.22 | 0.27 | 0.22 | 0.12 | 0.22 | 0.32 | **0.32** |
| W | | cintrl | Maximum interception storage of rain per unit LAI (mm). | 0.14 | 0.16 | 0.19 | 0.13 | 0.15 | 0.11 | 0.19 |
| W | | fxylem | Fraction of internal plant resistance to water flow that is in the xylem (-). | 0.17 | 0.16 | 0.18 | 0.10 | 0.16 | 0.15 | 0.18 |
| W | | mxkpl | Maximum internal conductivity for water flow through the plants (mm d^-1^). | 0.18 | 0.25 | 0.25 | 0.16 | 0.17 | 0.16 | **0.25** |
| W | | psicr | Critical leaf water potential at which stomates close (MPa). | 0.69 | 0.57 | 0.79 | 0.74 | 0.48 | 0.35 | **0.79** |
| W | | betaroot | Shape parameter for rootlength density depth distribution (-). | 0.49 | 0.47 | 0.54 | 0.28 | 0.45 | 0.31 | **0.54** |
| W | | maxrootdepth | Maximum root depth (-). | 0.31 | 0.38 | 0.37 | 0.18 | 0.25 | 0.16 | **0.38** |
| W | | maxlai | Maximum projected leaf area index (-). | 0.14 | 0.19 | 0.18 | 0.20 | 0.16 | 0.17 | **0.20** |
| W | | rssa | Soil evaporation resistance at field capacity (s m^-1^). | 0.16 | 0.21 | 0.20 | 0.23 | 0.17 | 0.16 | **0.23** |
| W | | ksat4 | Saturated hyraulic conductivity parameter of Mualem hydraulic conductivity function (mm d-1) in layer 4. | 0.16 | 0.18 | 0.19 | 0.11 | 0.14 | 0.15 | 0.19 |
| W | | ksat5 | Saturated hyraulic conductivity parameter of Mualem hydraulic conductivity function (mm d-1) in layer 5. | 0.15 | 0.13 | 0.17 | - | - | 0.13 | 0.17 |
| W | | ths4 | Saturation water content fraction in layer 4. | 0.16 | 0.13 | 0.18 | 0.15 | 0.12 | 0.13 | 0.18 |
| W | | ths5 | Saturation water content fraction in layer 5. | 0.18 | 0.13 | 0.09 | - | - | 0.10 | 0.18 |
| P | | alb | Albedo of soil/vegetation surface without snow (-). | 0.15 | 0.13 | 0.16 | 0.16 | 0.16 | 0.17 | 0.17 |
| P | | albsn | Albedo of soil/vegetation surface with snow (-). | 0.17 | 0.15 | 0.13 | 0.12 | 0.14 | 0.13 | 0.17 |
| P | | drain | Switch for lower boundary condition to be free drainage (1) or no flow (0). | 0.17 | 0.18 | 0.15 | 0.17 | 0.16 | 0.15 | 0.18 |
| P | | ilayer | Number of layers from top to which infiltration is distributed (-). | 0.21 | 0.23 | 0.20 | 0.14 | 0.31 | 0.16 | **0.31** |
| P | | infexp | Shape parameter for distribution of infiltration in first ILayer, for value 0 infiltration is in top layer only (-). | 0.17 | 0.15 | 0.15 | 0.11 | 0.16 | 0.19 | 0.19 |
| P | | bypar | Switch to allow (1) or prevent (0) bypass flow in deeper layers (-). | 0.13 | 0.17 | 0.17 | 0.15 | 0.17 | 0.17 | 0.17 |
| P | | glmax | Maximum leaf vapour conductance when stomata are fully open (m s^-1^). | 0.18 | 0.21 | 0.15 | 0.17 | 0.18 | 0.19 | **0.21** |
| P | | cvpd | Vapour pressure deficit at which leaf conductance is halved (kPa). | 0.18 | 0.17 | 0.14 | 0.19 | 0.14 | 0.23 | **0.23** |
| P | | r5 | Solar radiation level at which leaf conductance is half of its value at maximum shortwave radiation (W m^-2^). | 0.19 | 0.15 | 0.18 | 0.13 | 0.18 | 0.28 | **0.28** |
| P | cintrl | | Maximum interception storage of rain per unit LAI (mm). | 0.15 | 0.18 | 0.15 | 0.15 | 0.15 | 0.15 | 0.18 |
| P | fxylem | | Fraction of internal plant resistance to water flow that is in the xylem (-). | 0.15 | 0.14 | 0.20 | 0.17 | 0.13 | 0.17 | **0.20** |
| P | mxkpl | | Maximum internal conductivity for water flow through the plants (mm d^-1^). | 0.19 | 0.20 | 0.24 | 0.19 | 0.19 | 0.19 | **0.24** |
| P | psicr | | Critical leaf water potential at which stomates close (MPa). | 0.29 | 0.24 | 0.22 | 0.17 | 0.26 | 0.18 | **0.29** |
| P | betaroot | | Shape parameter for rootlength density depth distribution (-). | 0.33 | 0.50 | 0.38 | 0.28 | 0.32 | 0.22 | **0.50** |
| P | maxrootdepth | | Maximum root depth (-). | 0.27 | 0.42 | 0.27 | 0.26 | 0.22 | 0.17 | **0.42** |
| P | maxlai | | Maximum projected leaf area index (-). | 0.14 | 0.13 | 0.19 | 0.15 | 0.16 | 0.22 | **0.22** |
| P | rssa | | Soil evaporation resistance at field capacity (s m^-1^). | 0.17 | 0.18 | 0.13 | 0.12 | 0.19 | 0.15 | 0.19 |
| P | ksat4 | | Saturated hyraulic conductivity parameter of Mualem hydraulic conductivity function (mm d-1) in layer 4. | 0.13 | 0.19 | 0.18 | 0.25 | 0.23 | 0.15 | **0.25** |
| P | ksat5 | | Saturated hyraulic conductivity parameter of Mualem hydraulic conductivity function (mm d-1) in layer 5. | 0.12 | 0.21 | 0.14 | - | - | 0.14 | 0.21 |
| P | ths4 | | Saturation water content fraction in layer 4. | 0.17 | 0.16 | 0.14 | 0.10 | 0.15 | 0.15 | 0.17 |
| P | ths5 | | Saturation water content fraction in layer 5. | 0.26 | 0.18 | 0.12 | - | - | 0.16 | **0.26** |

Supplementary Table 2 Calibration and validation performance metric Nash-Sutcliff (NSE) - and Kling Gupta efficiency (KGE) for different soil depth and pedotransfer functions (PTF) with W referring to the PTF of Wessolek et al., 2009 and P to Puhlmann and Wilpert, 2011.

|  |  | calibration  2015-2017 | | calibration  2015-2018 | | temporal validation | spatial validation |
| --- | --- | --- | --- | --- | --- | --- | --- |
| PTF | depth | NSE | KGE | NSE | KGE | NSE | NSE |
| W | 20 cm | 0.52 | 0.67 | 0.53 | 0.68 | 0.62 | 0.29 |
| W | 80 cm | 0.55 | 0.68 | 0.61 | 0.69 | 0.66 | 0.41 |
| W | 110 cm | 0.37 | 0.54 | 0.37 | 0.54 | 0.56 | -8.51 |
| W | 150 cm | 0.26 | 0.5 | 0.26 | 0.5 | 0.37 | -9.29 |
| P | 20 cm | 0.27 | 0.39 | 0.39 | 0.42 | 0.32 | 0.05 |
| P | 80 cm | 0.21 | 0.41 | 0.36 | 0.52 | 0.53 | -3.67 |
| P | 110 cm | 0.02 | 0.21 | 0.08 | 0.37 | 0.66 | -45.01 |
| P | 150 cm | -0.73 | -0.08 | -0.23 | -0.14 | 0.38 | -43.91 |

Supplementary Table 3 Posterior parameters for different species and pedotransfer function (PTF) with W referring to the PTF of Wessolek et al., 2009 and P to Puhlmann and Wilpert, 2011.

| PTF | species | ilayer | infexp | drain | glmax | r5 | rssa | psicr | mxkpl | max-root-depth | beta-root | cvpd | f-xylem |
| --- | --- | --- | --- | --- | --- | --- | --- | --- | --- | --- | --- | --- | --- |
| W | beech | 4.3 | 0.30 | 0.59 | 0.0103 | 225 | 701 | -1.08 | 16.05 | -1.68 | 0.99 |  |  |
| W | oak | 4.4 | 0.34 | 0.47 | 0.0111 | 198 | 613 | -1.31 | 18.95 | -1.68 | 0.98 |  |  |
| W | spruce/pine | 5.9 | 0.60 | 0.51 | 0.0073 | 296 | 807 | -1.58 | 12.50 | -1.38 | 0.96 |  |  |
| W | spruce | 3.7 | 0.50 | 0.72 | 0.0076 | 139 | 760 | -0.89 | 9.49 | -1.31 | 0.97 |  |  |
| W | beech/oak | 6.9 | 0.50 | 0.57 | 0.0114 | 228 | 648 | -0.91 | 20.94 | -1.68 | 0.99 |  |  |
| W | pine | 3.4 | 0.38 | 0.46 | 0.0094 | 298 | 795 | -1.38 | 17.81 | -1.32 | 0.97 |  |  |
| P | beech | 6.7 | 0.22 |  |  |  | 692 | -3.07 |  | -1.71 | 0.97 | 2.16 | 0.61 |
| P | oak | 8.0 | 0.12 |  |  |  | 338 | -2.74 |  | -1.84 | 0.98 | 2.17 | 0.32 |
| P | spruce/pine | 6.8 | 0.25 |  |  |  | 929 | -1.54 |  | -1.64 | 0.96 | 1.58 | 0.64 |
| P | spruce | 5.4 | 0.54 |  |  |  | 663 | -0.91 |  | -1.77 | 0.97 | 1.48 | 0.68 |
| P | beech/oak | 9.4 | 0.53 |  |  |  | 626 | -2.09 |  | -1.67 | 0.98 | 2.35 | 0.32 |
| P | pine | 6.4 | 0.22 |  |  |  | 637 | -3.26 |  | -1.47 | 0.97 | 2.07 | 0.51 |


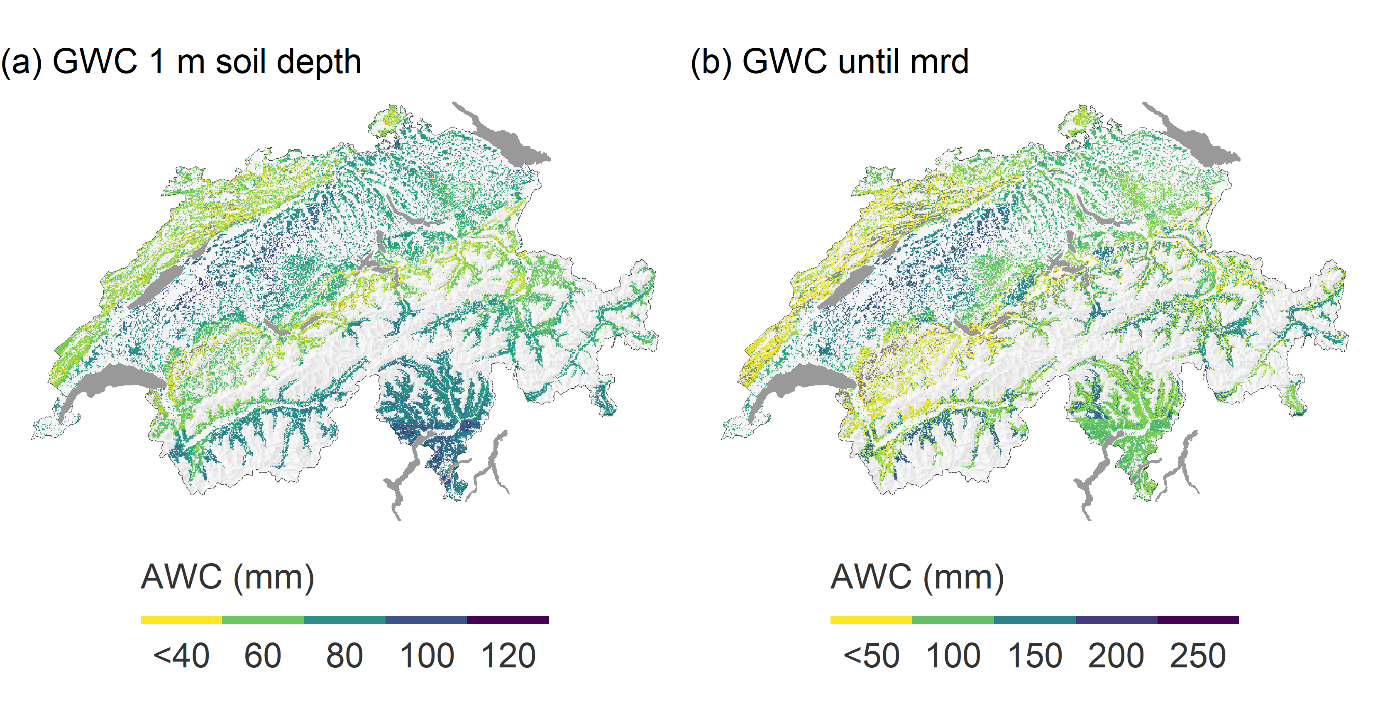


Supplementary Figure 1 Gravitational water capacity (mm) until 1 m soil depth and the maximum rooting depth (mrd).


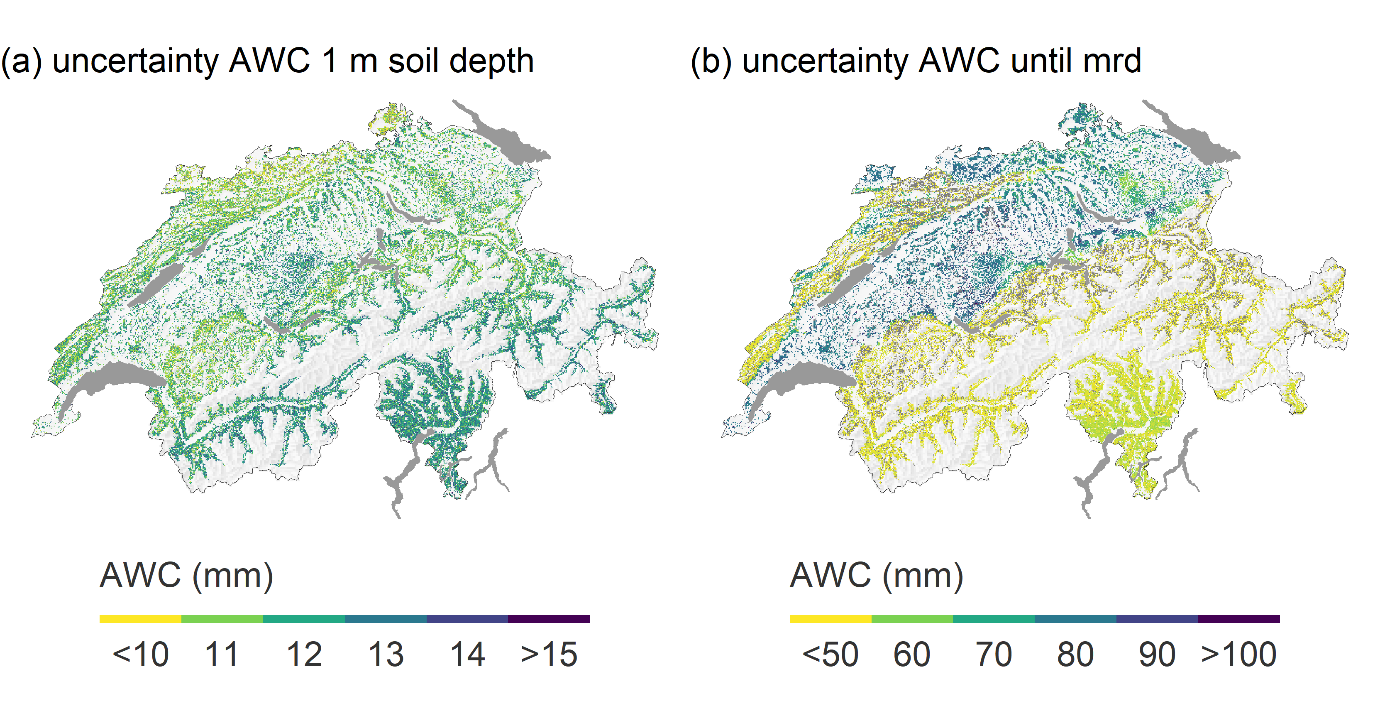


Supplementary Figure 2 Uncertainties of the available water storage capacity (AWC) until 1m soil depth and the mean maximum rooting depth (mrd).

Supplementary Figure 3 Average plant available - (AWC), gravitational (GWC) and residual (RES) water capacity per soil depth layer of the Swiss forest.
